# The spectraplakin Dystonin antagonizes YAP activity and suppresses tumourigenesis

**DOI:** 10.1038/s41598-019-56296-z

**Published:** 2019-12-27

**Authors:** Praachi B. Jain, Patrícia S. Guerreiro, Sara Canato, Florence Janody

**Affiliations:** 1 Instituto Gulbenkian de Ciência, Rua da Quinta Grande 6, P-2780-156 Oeiras, Portugal; 2 i3S - Instituto de Investigação e Inovação em Saúde, Universidade do Porto, Rua Alfredo Allen, 208, 4200-393 Porto, Portugal; 3 IPATIMUP - Instituto de Patologia e Imunologia Molecular da Universidade do Porto, Rua Júlio Amaral de Carvalho,45, 4200-135 Porto, Portugal

**Keywords:** Breast cancer, Cancer models, Cytoskeleton

## Abstract

Aberrant expression of the Spectraplakin Dystonin (DST) has been observed in various cancers, including those of the breast. However, little is known about its role in carcinogenesis. In this report, we demonstrate that Dystonin is a candidate tumour suppressor in breast cancer and provide an underlying molecular mechanism. We show that in MCF10A cells, Dystonin is necessary to restrain cell growth, anchorage-independent growth, self-renewal properties and resistance to doxorubicin. Strikingly, while Dystonin maintains focal adhesion integrity, promotes cell spreading and cell-substratum adhesion, it prevents Zyxin accumulation, stabilizes LATS and restricts YAP activation. Moreover, treating DST-depleted MCF10A cells with the YAP inhibitor Verteporfin prevents their growth. *In vivo*, the *Drosophila* Dystonin Short stop also restricts tissue growth by limiting Yorkie activity. As the two Dystonin isoforms BPAG1eA and BPAG1e are necessary to inhibit the acquisition of transformed features and are both downregulated in breast tumour samples and in MCF10A cells with conditional induction of the Src proto-oncogene, they could function as the predominant Dystonin tumour suppressor variants in breast epithelial cells. Thus, their loss could deem as promising prognostic biomarkers for breast cancer.

## Introduction

Breast cancer progression depends on cell autonomous regulatory mechanisms, driven by mutations and epigenetic changes, and on non-cell autonomous interactions with the surrounding tumour microenvironment^1,2^. During this multistep process, normal breast epithelial cells acquire various cellular properties arising from deregulated cellular signalling^3,4^. Among these, the highly conserved Hippo signal transduction pathway, originally discovered in the fruit fly *Drosophila melanogaster*, has emerged as a critical tumour suppressor pathway in breast cancer^5,6^. In mammals, the canonical Hippo pathway consists of the MST (sterile 20-like kinase) kinases, which activate the LATS1/2 (large tumour suppressor, Warts-Wts in *Drosophila*) kinases through phosphorylation^7^. In turn, LATS phosphorylates the transcriptional co-activators YAP and TAZ (Yorkie-Yki in *Drosophila*), thereby, limiting their nuclear import^8–15^. In contrast, when the Hippo pathway is deactivated, YAP and TAZ translocate into the nucleus where they drive gene expression in complex with transcription factors, such as TEA-domain containing sequence specific transcription factors (TEAD), promoting cell growth and tumourigenesis^16–21^. A plethora of architectural and mechanical cues, including extracellular matrix (ECM) stiffness, cell-cell adhesion, cell-matrix adhesion, cell density, cell shape and cell polarity, regulate this pathway^6^. Many of these regulatory inputs converge on the actin cytoskeleton to regulate YAP/TAZ/Yki^22–25^. Among these, the actin-associated LIM protein Zyxin antagonizes the effect of the FERM-domain protein Expanded on apical F-actin accumulation and on Yki-mediated tissue growth, as well as potentiates YAP/TAZ/Yki activity by destabilizing LATS/Warts in MDA-MB-231 cells and *Drosophila* epithelia^26–28^. Yet, a full and comprehensive understanding of the detailed molecular mechanisms linking upstream regulatory inputs, the cytoskeleton and Hippo signalling activity still remains elusive.

The cytoskeleton comprises three main elements, actin, intermediate filaments and microtubules. Together, they support a large number of cellular processes, including signalling, intracellular trafficking, polarity, migration, adhesion, cell division, mechanical strength and cellular shape^29^. Spectraplakins are giant cytolinkers, which have the rare ability to bind to all three main cytoskeletal elements and with transmembrane proteins to coordinate cytoskeletal dynamics. In mammals, two genes are known to encode for spectraplakins: microtubule and actin crosslinking factor 1 (*MACF1*; also known as actin crosslinking factor 7, *ACF7*) and Dystonin (*DST*; also known as bullous pemphigoid antigen 1, BPAG1)^30^. They are evolutionary conserved proteins and give rise to differentially spliced variants resulting in distinct isoforms. All annotated proteins contain a plakin domain, which associates with membrane-associated proteins. Additionally, these isoforms contain other molecular domains that mediate the interaction with distinct cytoskeletal elements. These domains include Calponin-homology domains (CH), which bind to actin filaments, a plakin repeat region, which links to intermediate filaments (IF-BD), a growth-arrest specific 2-related (GAR) microtubule-binding domain, an EF-hand calcium-binding domain, and a spectrin-repeat rod^30,31^.

While MACF1 appears to act as a potential oncoprotein, the role of DST in cancer is still unclear^32^. Microarray profiling indicates that DST is downregulated in the mammary epithelial cell line MCF10A with conditional Src induction (MCF10A-ER-Src), in Estrogen receptor (ER)-positive ductal carcinoma *in situ* (DCIS) and in invasive ductal carcinoma (IDC), irrespective of the ER status^33,34^. Consistent with a role of DST as a candidate tumour suppressor in breast cancer, the unique *Drosophila* DST Short stop (Shot) restricts Src-induced epithelial overgrowth and is required to restrain growth in wild type epithelia^33^. Accordingly, DST inhibits the tumourigenicity and invasion of DCIS.COM cells^35^. In contrast, in oral squamous cell carcinoma cells, the shorter DST isoform BPAG1e promotes migration, invasion and tumorigenic potential^36,37^.

Here, we provide a molecular mechanism for the tumour-suppressing function of DST. Our observations are consistent with a model by which DST restrains cellular transformation by hindering Zyxin accumulation, stabilizing LATS and preventing YAP activity in MCF10A cells and in *Drosophila* epithelia. As the tumour suppressor function of DST involves the shorter BPAG1eA and/or BPAG1e isoforms, they could be used as prognostic biomarkers for breast cancer.

## Results

### DST limits the growth of MCF10A cells with conditional Src activation

**Figure 1:**
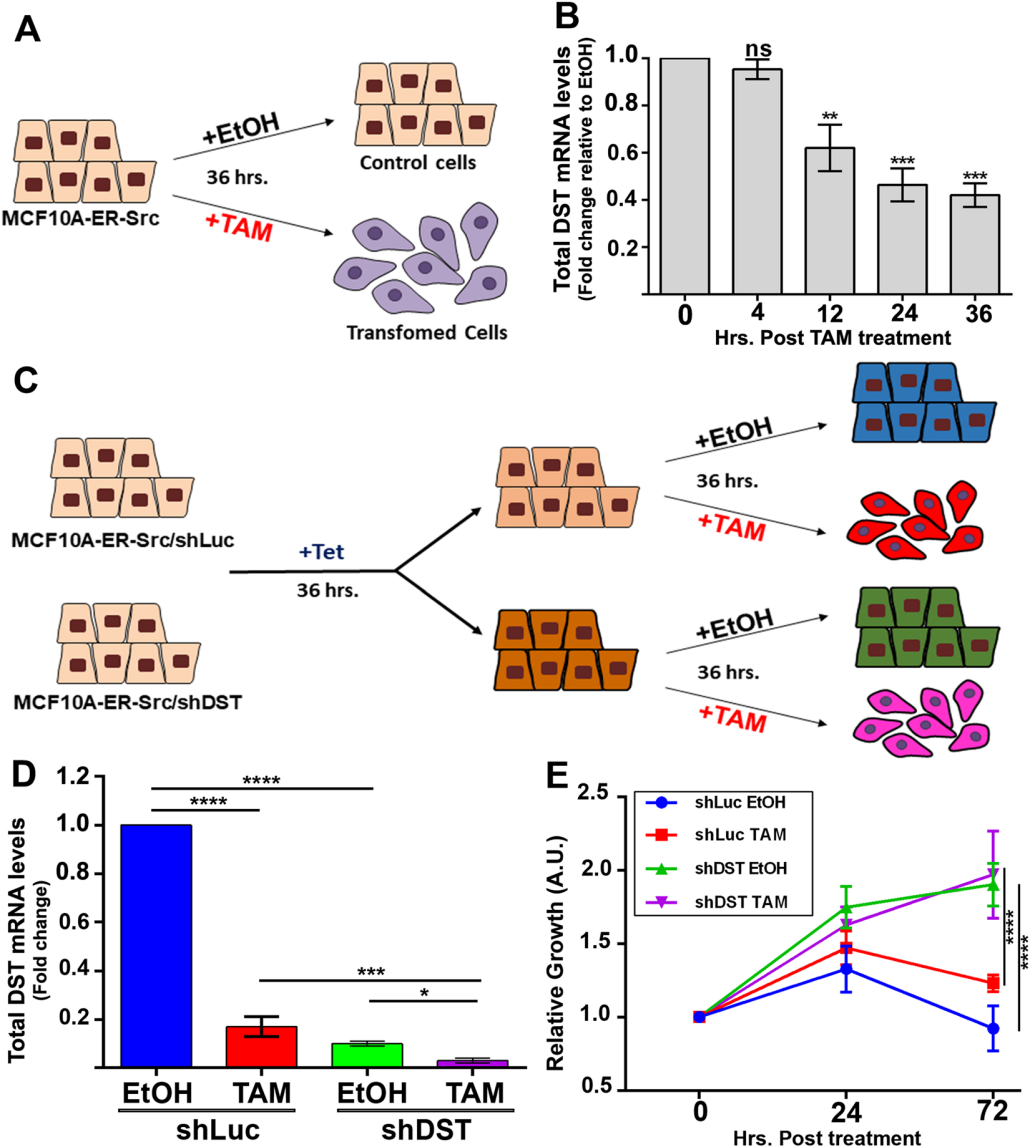
DST is downregulated by Src and limits Src-induced cell growth. **(A)** Schematic of the experimental design to analyse the effect of Src activation on DST mRNA levels. In contrast to MCF10A-ER-Src cells treated with EtOH, those treated with TAM for 36 hours acquire transformed features^33,38,39^. **(B)** Ratio of total DST mRNA levels between TAM- and EtOH-treated MCF10A-ER-Src cells for the same time points (0, 4, 12, 24 and 36 hours), normalized to GAPDH. Data are from three biological replicates performed in triplicates. **(C)** Schematic of the experimental design to analyse the effect of reducing further DST levels in MCF10A cells with conditional Src induction. MCF10A-ER-Src cells stably transfected with shDST or shLuc were treated with Tet for 36 hours and with EtOH or TAM for an additional 36 hours. **(D)** Total DST mRNA levels on extracts from MCF10A-ER-Src/shLuc or MCF10A-ER-Src/shDST treated with Tet for 36 hours and with EtOH or TAM for an additional 36 hours. Data are from three biological replicates performed in triplicates. **(E)** Time course of growth rate for MCF10A-ER-Src/shLuc or MCF10A-ER-Src/shDST before treatment (0 hours post-treatment) or treated with Tet for 24 hours (24 hours post-treatment) or treated with Tet for 36 hours and with EtOH or TAM for an additional 36 hours (72 hours post-treatment). A.U. Arbitrary Unit. Data are from three biological replicates performed in triplicates. For all quantifications, error bars indicate SD; ns indicates non-significant; *indicates P < 0.05; **indicates P < 0.005; ***indicates P < 0.001; ****indicates P < 0.0001.

To understand the contribution of DST in breast cancer cells, we first confirmed that transformation of the inducible MCF10A-ER-Src cell line was associated with the downregulation of DST. This cell line contains a fusion between v-Src and the ligand-binding domain of the ER^38,39^. Treatment of these cells with tamoxifen (TAM) induces a step wise increase in Src activation and the acquisition of transformed features within 36 hours^33,38^. MCF10A-ER-Src cells treated with TAM or with the vehicle EtOH were tested for DST mRNA levels at different time during the 36 hours of treatments (see experimental design in Fig. 1A), using primers amplifying all DST isoforms. The ratio of DST mRNA levels between cells treated with TAM and EtOH indicated that DST levels were significantly reduced by 38% 12 hours after treatment, and dropped by 58% at 36 hours (Fig. 1B). MCF10A-ER-Src cells in which we forced the expression of DST using the Clustered Regularly Interspaced Short Palindromic Repeats (CRISPR)-based activation system^40^ were unable to grow. Thus, to determine if the downregulation of DST was required for Src-induced cellular transformation, we tested whether further reducing DST levels potentiates the growth of TAM-treated MCF10A-ER-Src cells. MCF10A-ER-Src cells were stably transfected with Tetracycline (Tet)-inducible short-hairpin RNA (shRNA) against all DST isoforms (MCF10A-ER-Src/shDST) or against Luciferase (MCF10A-ER-Src/shLuc). Cells were then exposed to Tet for 36 hours before being treated with TAM or with the vehicle EtOH for an additional 36 hours (Fig. 1C). Tet decreased DST mRNA levels by 9 folds in EtOH-treated MCF10A-ER-Src/shDST cells compared to those carrying shLuc. Moreover, it further reduced DST levels by 5.6 folds in TAM-treated MCF10A-ER-Src/shDST cells compared to those expressing shLuc (Fig. 1D). Consistent with a role of DST in preventing Src-induced cellular transformation, further reducing DST levels in TAM-treated cells significantly increased cell growth (Fig. 1E). Importantly, in control EtOH-treated cells, knocking down DST also enhanced cell growth (Fig. 1E). Taken together, these observations suggest a role of DST in preventing the growth of MCF10A-ER-Src cells with Src overactivation and of untransformed MCF10A cells.

### DST is necessary to prevent transformation in MCF10A cells

**Figure 2:**
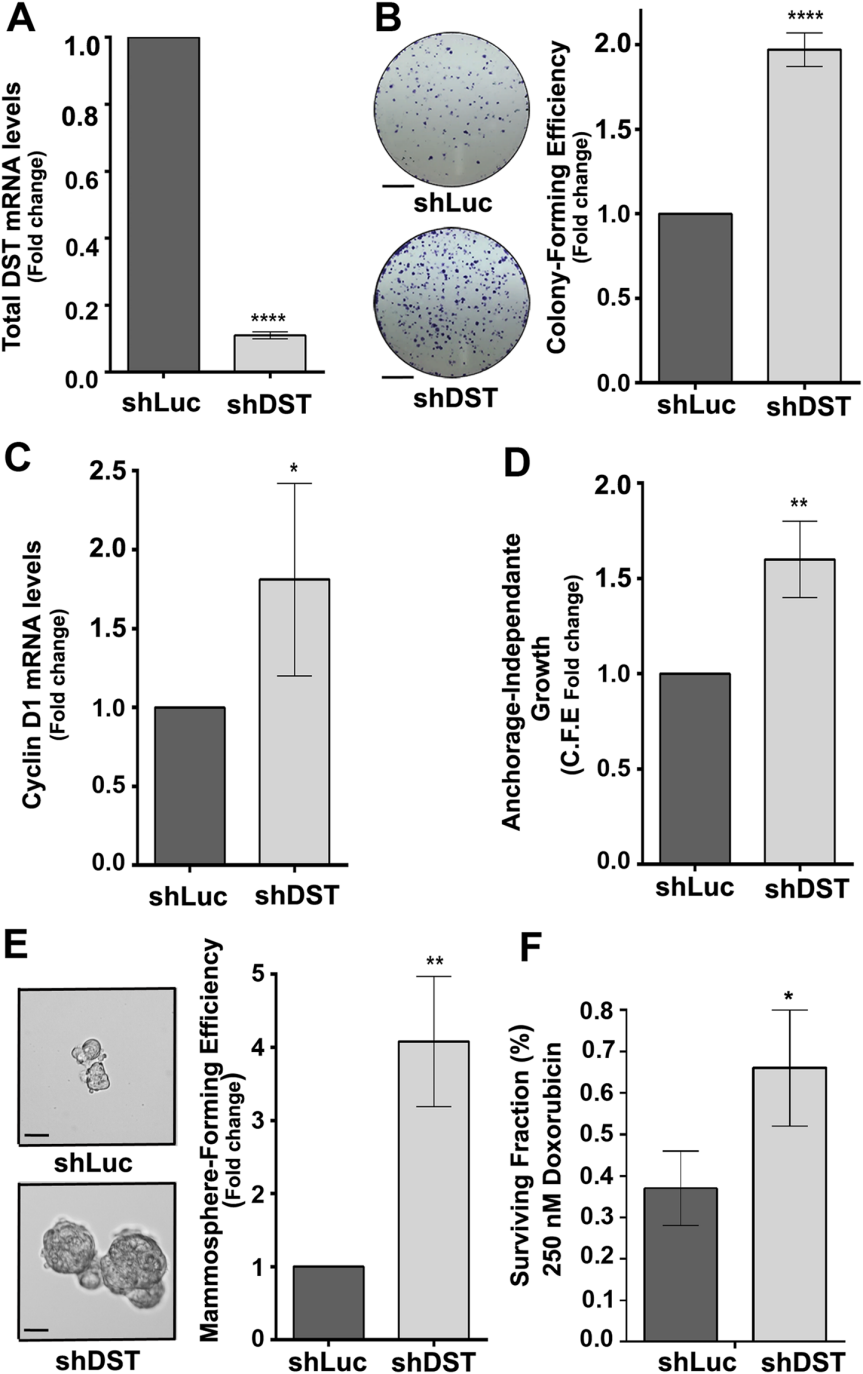
Knocking down DST is sufficient to induce the acquisition of transformed features to MCF10A cells. **(A)** Fold change of total DST mRNA levels between shLuc- and shDST-expressing MCF10A cells, normalized to GAPDH. Data are from four biological replicates performed in triplicates. **(B)** (left panels) Representative images of colonies from shLuc- or shDST-expressing MCF10A cells grown in clonogenic assays. Scale bars represent 5 mm. (Right panel) Fold change in colony-forming efficiency between shLuc- and shDST-expressing MCF10A cells. Data are from three biological replicates performed in triplicates. **(C)** Fold change in Cyclin D1 mRNA levels between shLuc- and shDST-expressing MCF10A cells. Data are from four biological replicates performed in triplicates. **(D)** Fold change in colony-forming efficiency (C.F.E.) between shLuc- and shDST-expressing MCF10A cells grown in soft agar. Data are from three biological replicates performed in triplicates. **(E)** (Left panels) Representative images of shLuc- or shDST-expressing MCF10A mammospheres. Scale bars represent 50 μm. (Right panel) Fold change in mammosphere-forming efficiency between shLuc- and shDST-expressing MCF10A cells. Data are from three biological replicates performed in triplicates. **(F)** Percentage (%) of surviving shLuc- or shDST-expressing MCF10A cells, treated with 250 nM of Doxorubicin. Data are from three biological replicates performed in triplicates. For all quantifications, error bars indicate SD.; *indicates P < 0.05; **indicates P < 0.005; ****indicates P < 0.0001.

To determine if DST acts as a *bona-fide* tumour suppressor, we generated stable MCF10A cells carrying Tet-inducible shRNA against all DST isoforms (shDST). Treating these cells with Tet reduced DST mRNA levels by 90% compared to control Tet-treated MCF10A-shLuc (shLuc) cells (Fig. 2A). Knocking down DST was sufficient to increase the colony-forming ability of MCF10A cells in clonogenic assays (Fig. 2B) and significantly upregulated Cyclin D1 mRNA levels (Fig. 2C), a known regulator of G1 to S phase progression^41^. Moreover, Tet-treated shDST cells produced significantly higher number of anchorage-independent colonies in soft agar compared to those expressing shLuc cells (Fig. 2D). Furthermore, knocking down DST also increased the mammosphere-forming abilities of MCF10A cells, as well as mammospheres size that were on average 1.4 times bigger (Fig. 2E). Finally, depleting DST conferred cells with significantly increased survival potential upon exposure to 250 nM of Doxorubicin (Fig. 2F), suggesting that the downregulation of DST promotes chemoresistance to these cells. To discard the possibility that the transformed phenotype of Tet-treated shDST cells resulted from off-target effects, we generated stable MCF10A cells expressing independent Tet-inducible shRNA against all DST isoforms (shDST#2). Treatment of these cells with Tet reduced DST mRNA levels by 55% (Supplementary Fig. S1), similar to Src’s effect on DST levels in MCF10A-ER-Src cells (Fig. 1B). This was sufficient to promote the acquisition of transformation features, as Tet-treated shDST#2 cells showed increased abilities to grow as colonies in clonogenic assays and to form mammospheres compared to those expressing shLuc (Supplementary Fig. S1). All together, we conclude that the presence of DST is necessary in MCF10A cells to restrict the gain of transformed features.

### DST promotes cell-substrate adhesion and prevents Zyxin accumulation

**Figure 3:**
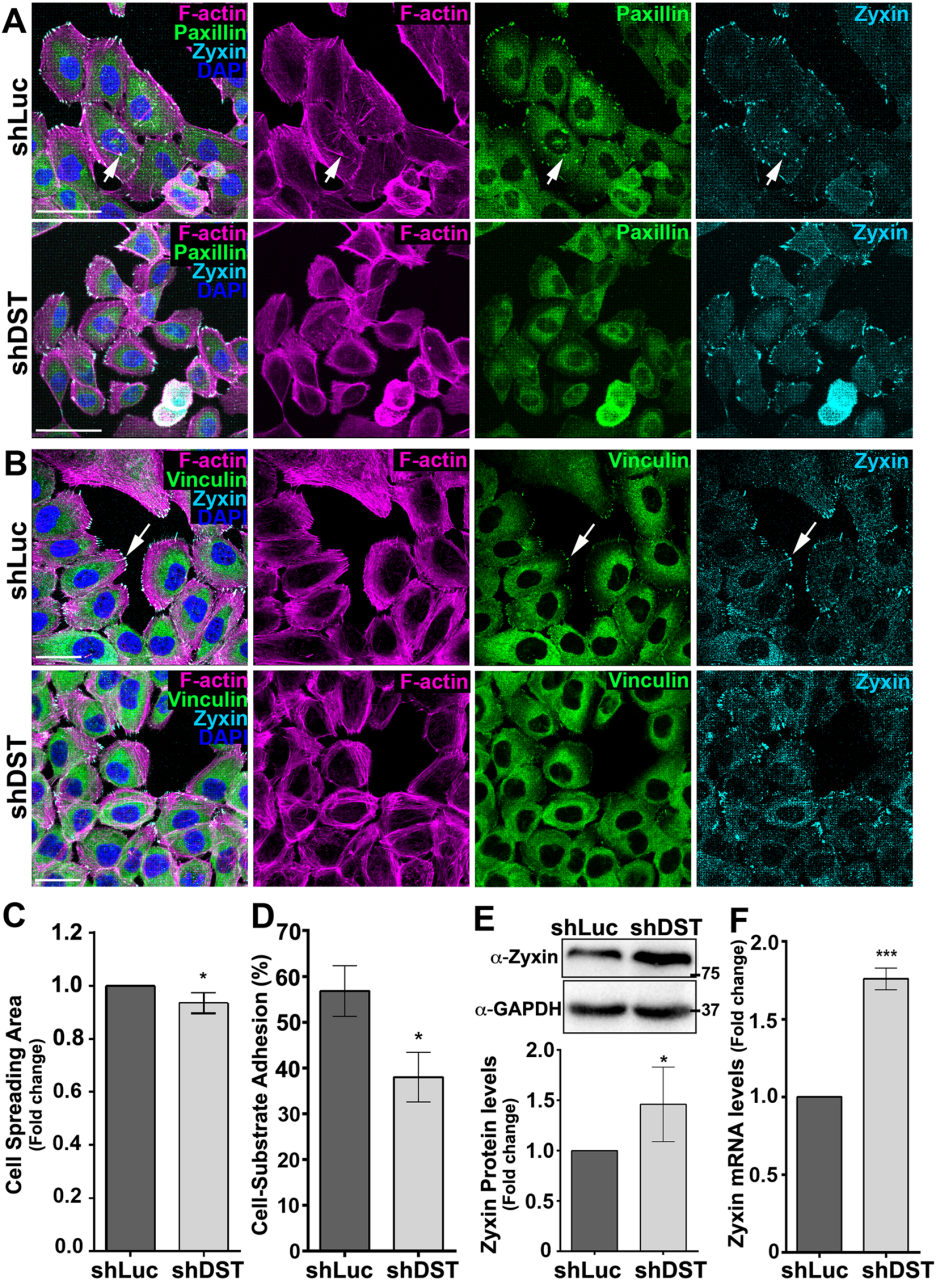
Knocking down DST affects FAs and cell-substratum adhesion. **(A,B)** Standard confocal sections of shLuc- or shDST-expressing MCF10A cells, stained with Phalloidin (Magenta) to mark F-actin, DAPI (Blue) to mark nuclei, anti-Zyxin (Cyan blue) and **(A)** anti-Paxillin (Green) or **(B)** anti-Vinculin. Scale bars represent (**A**) 50 µm or (**B**) 30 µm. **(C)** Fold change in cell spreading area between shLuc- and shDST-expressing MCF10A cells. Data are from three biological replicates performed in triplicates. A total of 1099 and 948 shLuc- and shDST-expressing cells, respectively, were quantified **(D)** Percentage (%) of shLuc- or shDST-expressing MCF10A cells adhering to the substratum. Data are from three biological replicates performed in triplicates. **(E)** (Upper panels) Western blots on protein extracts from shLuc- or shDST-expressing MCF10A cells, blotted with anti-Zyxin (upper bands) or anti-GAPDH (lower bands). (Lower panels) Ratio of Zyxin levels between shLuc- and shDST-expressing MCF10A cells, normalized to GAPDH. Data are three four biological replicates. **(F)** Fold change in Zyxin mRNA levels between shLuc- and shDST-expressing MCF10A cells. Data are from three biological replicates, performed in triplicate. For all quantifications, error bars indicate SD.; *indicates P < 0.05; ***indicates P < 0.001.

We then asked if MCF10A cells knocked down for DST gained morphological features of transformed cells. Strikingly, sub-confluent shDST-expressing MCF10A cells were significantly less spread than control cells expressing shLuc (Fig. 3A–C). In addition, while control cells contained Paxillin (Fig. 3A) and Vinculin- (Fig. 3B) positive focal adhesions (FAs) associated with dorsal actin stress fibres (arrows in Fig. 3A), shDST-expressing cells failed to concentrate Paxillin and Vinculin into spikes, did not show obvious ventral stress fibres, while displayed a dense actin filament meshwork at the cell edge (Fig. 3A,B). Moreover, Tet-treated shDST cells exhibited decreased ability to adhere to the substrate (Fig. 3D). Thus, DST maintains FA integrity and promotes cell spreading. However, Zyxin, another major component of FAs^42^, was still accumulated in shDST-expressing cells (Fig. 3A,B). Quantification of the levels of Zyxin protein (Fig. 3E and Supplementary Fig. S2) and mRNA (Fig. 3F) showed higher levels in cells knocked down for DST. In contrast, the levels of phosphorylated Src, a known regulator of FAs assembly^43^, was not affected in these cells (Supplementary Fig. S1 and S2). Taken together, we conclude that reducing DST function affects FA integrity, decreases cell-substrate adhesion and promotes Zyxin accumulation.

### DST restricts YAP activity

**Figure 4:**
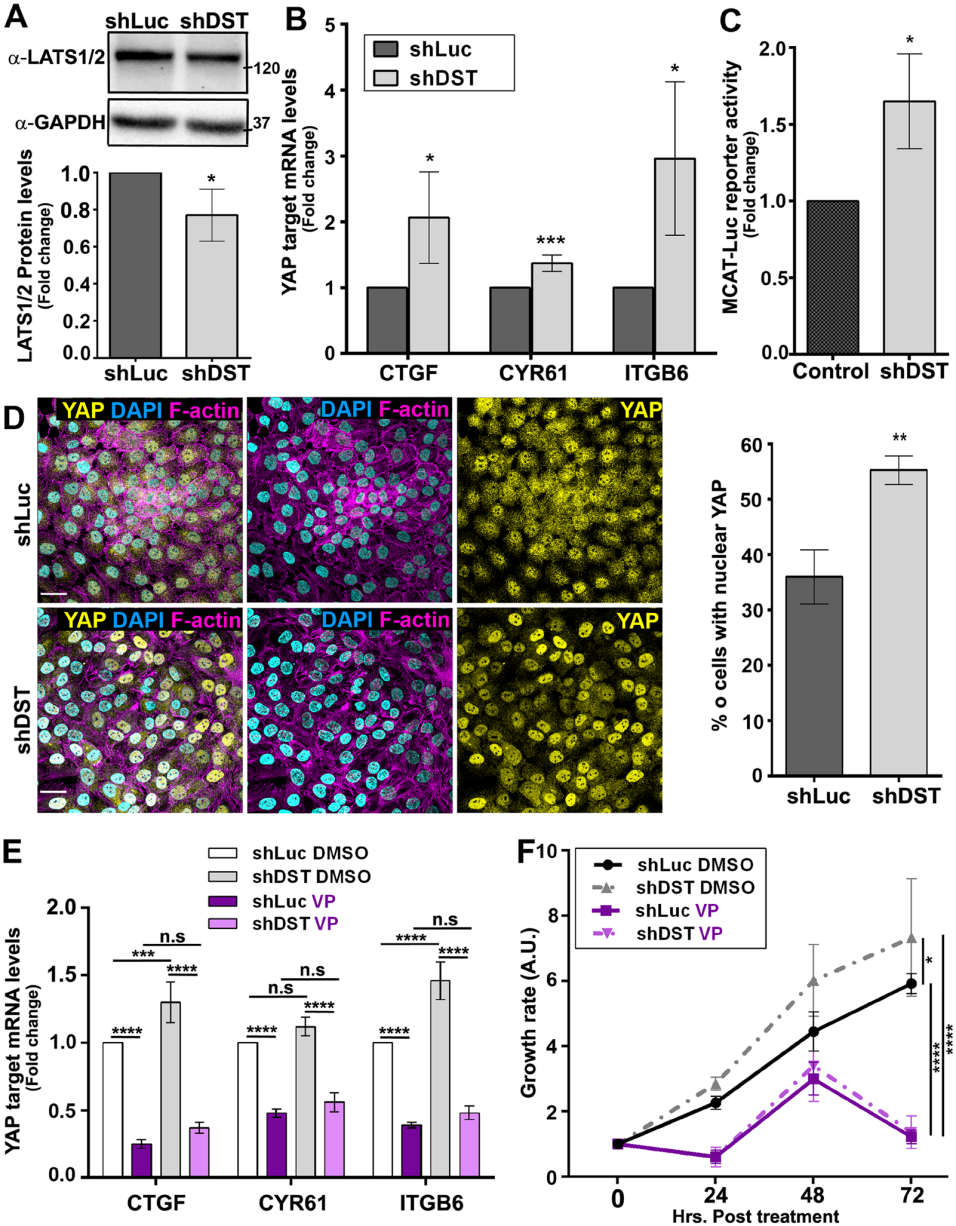
Knocking down DST enhances YAP activity. **(A)** (Upper panels) Western blots on protein extracts from shLuc- or shDST-expressing MCF10A cells, blotted with anti-LATS1/2 (upper bands) or anti-GAPDH (lower bands). (Lower panels) Ratio of LATS1/2 levels between shLuc- and shDST-expressing MCF10A cells, normalized to GAPDH. Data are from three biological replicates. **(B)** Fold changes in CTGF or CYR61 or ITGB6 mRNA levels between shLuc- and shDST-expressing MCF10A cells. Data are from four biological replicates, performed in triplicates. **(C)** Fold changes in Luciferase activity between untreated MCF10A-shLuc cells and Tet-treated MCF10A-shDST cells, transfected with the YAP/TAZ-responsive MCAT-Luc reporter gene. Data are from three biological replicates, performed in triplicates. **(D)** (Left panels) Standard confocal sections of shLuc- or shDST-expressing MCF10A cells, stained with Phallodin (Magenta) to mark F-actin, anti-YAP (Yellow) and anti-Lamin (Blue) to mark the nuclear membrane. Scale bars represent 30 µm. (Right panel) quantifications of the percentage (%) of shLuc- or shDST-expressing MCF10A cells in which the ratio of YAP staining is higher in the nucleus than in the cytoplasm. Data are from three biological replicates. A total of 775 and 809 shLuc- and shDST-expressing cells, respectively, were quantified blind twice. **(E)** Fold changes in CTGF or CYR61 or ITGB6 mRNA levels in shLuc- or shDST-expressing MCF10A cells, treated with DMSO or Verteporfin (VP). Data are from three biological replicates, performed in triplicates. **(F)** Time course of growth rate for shLuc- or shDST-expressing MCF10A cells, before treatment (0 hours post-treatment) or treated with DMSO or VP for 24, 48 or 36 hours. A.U. Arbitrary Unit. Data are from three biological replicates. For all quantifications, error bars indicate SD.; ns indicates non-significant; *indicates P < 0.05; **indicates P < 0.005; ***indicates P < 0.001; ****indicates P < 0.0001.

Zyxin is known to induce YAP/Yki activity by destabilizing LATS/Wts and by linking F-actin regulation to Yki-mediated tissue growth^26–28^. We therefore tested whether DST prevents cellular transformation through the regulation of Hippo pathway activity. Accordingly, LATS levels were significantly lower in Tet-treated shDST cells compared to those expressing shLuc (Fig. 4A and Supplementary Fig. S2). Moreover, qRT-PCR revealed that cells knocked down for DST expressed higher levels of the YAP/TAZ target genes Connective Tissue Growth Factor (CTGF), Cysteine rich angiogenic inducer 61 (CYR61) and Integrin β6 (ITGB6) (Fig. 4B). To confirm the effect of DST on YAP/TAZ transcriptional activity, we analysed the activity of a MCAT (muscle C, A and T)-dependent luciferase reporter (MCAT-Luc), which contains TEAD binding sites and responds to YAP/TAZ activity^44^. Tet-treated shDST cells showed higher levels of Luciferase activity compared to those grown in the absence of Tet (Fig. 4C). Furthermore, knocking down DST significantly increased the percentage of cells with YAP localized predominantly in the nucleus (Fig. 4D). Thus, DST stabilizes LATS protein levels, limits YAP translocation into the nucleus and the upregulation of YAP target genes.

We then tested if YAP potentiates the growth of DST-depleted cells by treating cells with the YAP inhibitor Verteporfin (VP), which blocks the interaction between YAP and the transcription factor TEAD, therein repressing YAP’s function^45^. Consistent with a role of YAP in promoting the transformation of DST-depleted cells, VP treatment downregulated CTGF, CYR61 and ITGB6 in both, shLuc- and shDST-expressing cells, compared to those treated with DMSO (Fig. 4E). Moreover, VP treatment reduced the growth rate of Tet-treated shLuc- and shDST cells (Fig. 4F). These observations suggest that DST prevents cellular transformation by limiting YAP activity.

### *In vivo*, the *Drosophila* DST Shot restricts tissue growth by limiting Yki activity

**Figure 5:**
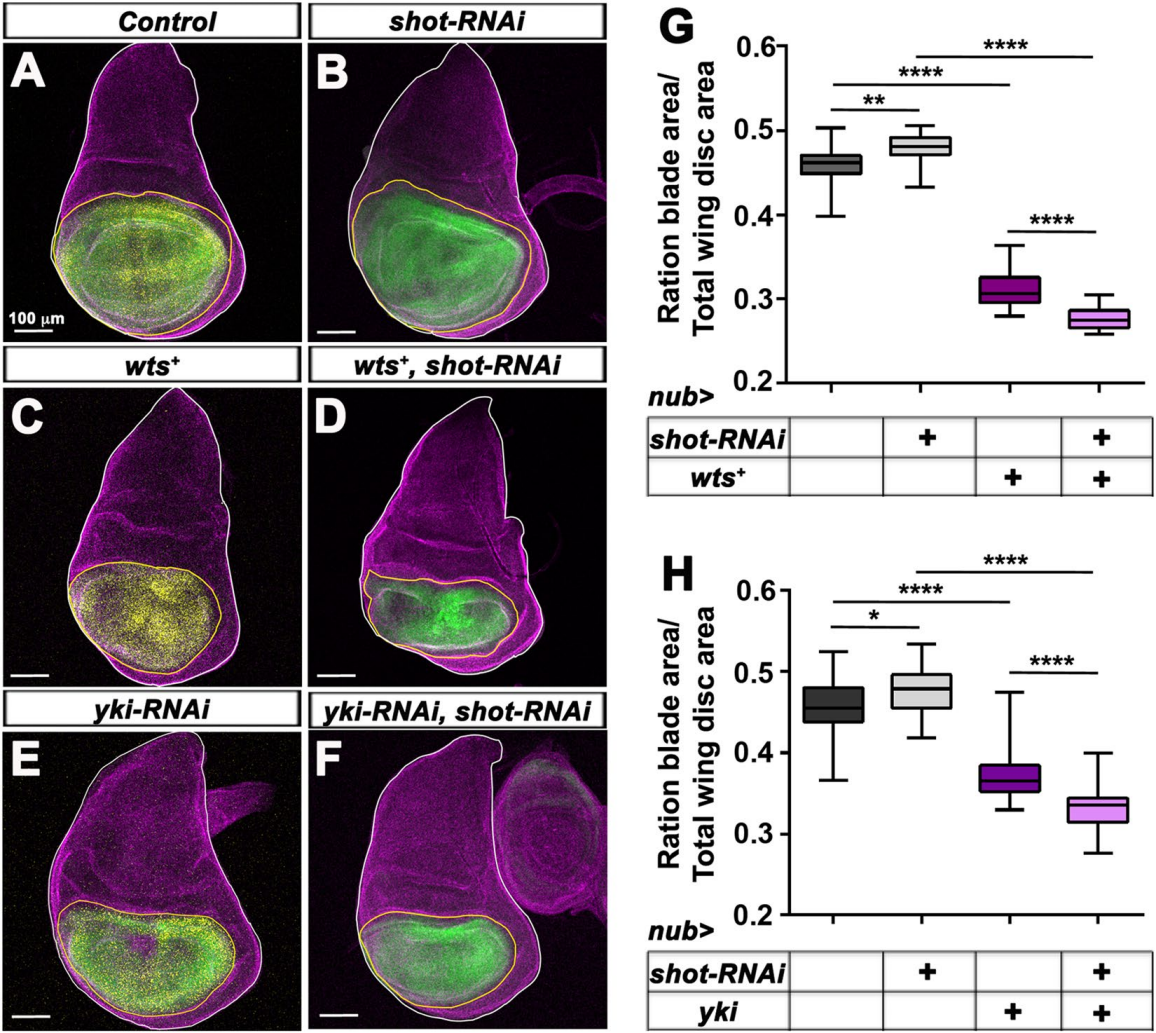
Overexpressing *wts* or knocking down *yki* suppresses the overgrowth of distal wing discs knocked down for *shot*. **(A–F)** standard confocal sections of third instar wing imaginal discs from females with dorsal up in which *nub-*Gal4 drives **(A)** UAS-*GFP* (green), UAS-*mCherry* (yellow) and UAS-*RFP* (yellow) or **(B)** two copies of UAS-*GFP* (green) and UAS-*shot-RNAi* or **(C)** UAS-*mCherry* (yellow), UAS-*RFP* (yellow) and UAS-*Myc::wts* (wts+) or **(D)** UAS-*GFP* (green), UAS-*shot-RNAi* and UAS-*Myc::wts* (wts+) or **(E)** UAS-*GFP* (green), UAS-*mCherry* (yellow) and UAS-*yki-RNAi* or **(F)** UAS*-GFP* (green), UAS*-shot-RNAi* and *UAS-yki-RNAi*. Discs are stained with anti-Disc large (Dlg) (magenta) to outline the disc area. The white lines outline the whole wing disc area. The yellow lines outline the *nub* > *GFP-* and/or *nub* > *mCherry; RFP-*expressing domains. Scale bars represent 100μm. **(G,H)** Quantification of the ratio between the *GFP-* or *mCherry*-expressing area and the total wing disc area in discs expressing the indicated UAS constructs under *nub-*Gal4. **(G)** 23 samples were analysed for *nub* > *GFP, mCherry, RFP*. 28 samples were analysed for *nub* > *2XGFP, shot-RNAi*. 23 samples were analysed for *nub* > *mCherry, RFP, wts*^+^. 20 samples were analysed for *nub* > *GFP, shot-RNAi, wts*^+^. **(H)** 24 samples were analysed for *nub* > *GFP, mCherry, RFP*. 28 samples were analysed for *nub* > *2XGFP, shot-RNAi*. 30 samples were analysed for *nub* > *GFP, mCherry, yki-RNAi*. 28 samples were analysed for *nub* > *GFP, shot-RNAi, yki-RNAi*. Error bars indicate SD. *indicates P < 0.05, **indicates P < 0.001, ****indicates P < 0.0001.

DST is evolutionarily well conserved between human and *Drosophila*^31^. Reminiscent to DST’s effect on limiting the growth of MCF10A cells, expressing double strand RNA (dsRNA) directed against the *Drosophila* DST orthologue Shot (*shot-RNAi*) is sufficient to induce overgrowth in the distal wing imaginal disc epithelium. Conversely, overexpressing the full-length *Shot-PE* isoform fused to GFP (*ShotL(A)::GFP*), which can fully or partially rescue all *shot* mutant phenotypes tested so far^46^, reduces distal wing disc growth^33^. To determine if Shot prevents tissue overgrowth by limiting the activity of the YAP orthologue Yki *in vivo*, we analysed the effect of overexpressing *wts* or of knocking down *yki* on the overgrowth of distal wing discs carrying UAS-*shot-RNAi* expressed under *nubbin-* (*nub*)-Gal4 control. As the expression of UAS transgenes can vary based on the number of Gal4 transcriptional activator available per UAS transgenes, experiments were performed ensuring that all genetic backgrounds contained the same number of UAS transgenes, which were normalized using UAS-*GFP*, UAS-*mCherry* and UAS-*RFP.* As reported previously^33^, knocking down *shot* increased significantly the ratio between the *nub* > *GFP*-expressing area and the total wing disc area (Fig. 5B,G,H), as compared to control discs expressing *GFP, mCherry* and *RFP* (Fig. 5A,G,H). Overexpressing *wts* (*wts*^+^) (Fig. 5D,G) or knocking down *yki* (*yki-RNAi*) (Fig. 5F,H) in *nub* > *shot-RNAi-*expressing discs drastically prevented the overgrowth of these tissues, which were even smaller that wing discs overexpressing *wts* (Fig. 5C,G) or *yki-RNAi* (Fig. 5E,H). Thus, the overgrowth of *shot*-depleted wing discs is dependent on Yki activity.

**Figure 6:**
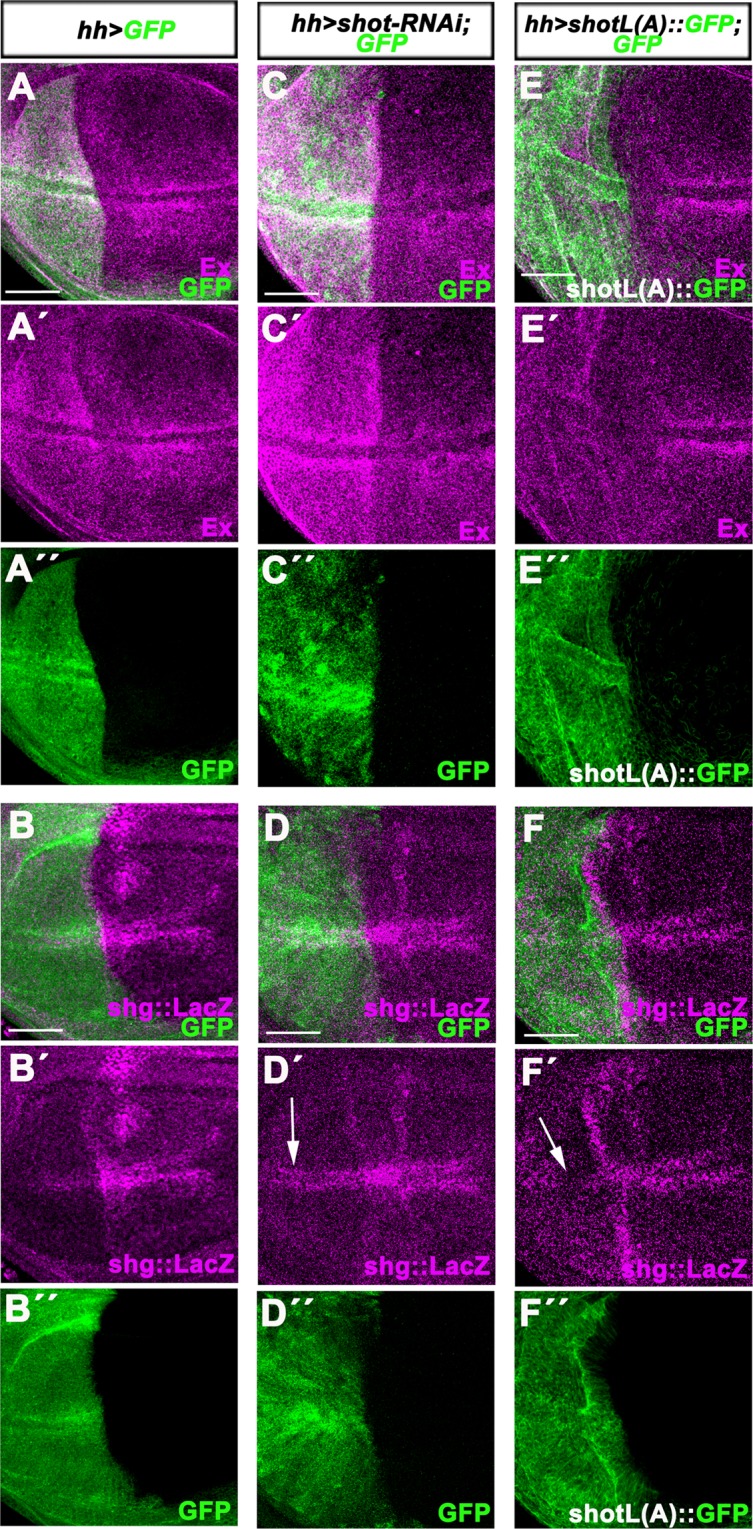
Knocking down or overexpressing *shot* in the distal wing disc epithelium affects the expression of Yki target genes. All panels show standard confocal sections of third instar wing imaginal discs with posterior to the left and dorsal up in which *hh-*Gal4 drives **(A-A**″**,B-B**″**)** UAS-*GFP* (green in **A**,**A′,B,B′**) or **(C-C**″**,D-D**″**)** UAS-*GFP* (green in **C**,**C″,** **D**,**D″**) and UAS-*shot-RNAi* or **(E-E**″**,F-F**″**)** UAS-*shotL(A)::GFP* (green in **E,E″**,**F,F″**). Discs are stained with (**A-A**′**,C-C**′**,E-E**′) anti-Ex (magenta) or (**B-B′,D-D′,F-F′**) anti-β.galactosidade to reveal *shg-LacZ* expression (magenta). The white arrows in (**D**′ and **F**′) indicate the upregulation or downregulation, respectively, of *shg::LacZ* in cells flanking the dorsal-ventral boundary. Scale bars represent 50 μm.

We then tested if expressing *shot-RNAi* or overexpressing *ShotL(A)::GFP* affects the expression of Yki target genes in the posterior wing disc compartment using the *hedgehog-*Gal4 (*hh-*Gal4) driver, thus allowing comparison to the wild type anterior compartment. In control *GFP*-expressing wing discs, the Yki target gene Expanded (Ex) was found accumulated at higher levels in the posterior compartment expressing *GFP* only (Fig. 6A-A″). In contrast, a *LacZ* enhancer trap insertion into the *shotgun* gene (*shg*-LacZ), another Yki target^47^, was expressed at lower levels in the *GFP*-expressing domain (Fig. 6B-B″). Expressing *shot-RNAi* and *GFP* with *hh-*Gal4 further potentiated Ex accumulation (Fig. 6C-C″) and enhanced *shg-*LacZ expression (Fig. 6D-D″) in the posterior wing disc compartment. In contrast, overexpressing *shotL(A)::GFP* with *hh-*Gal4 reduced both, Ex protein levels (Fig. 6E-E″) and *shg-*LacZ expression (Fig. 6F-F″). Thus, reminiscent to DST function in MCF10A cells, in the distal *Drosophila* wing disc epithelium, Shot also restricts the expression of Yki target genes.

### BPAG1e and BPAG1eA are downregulated by Src and in breast tumour samples

**Figure 7:**
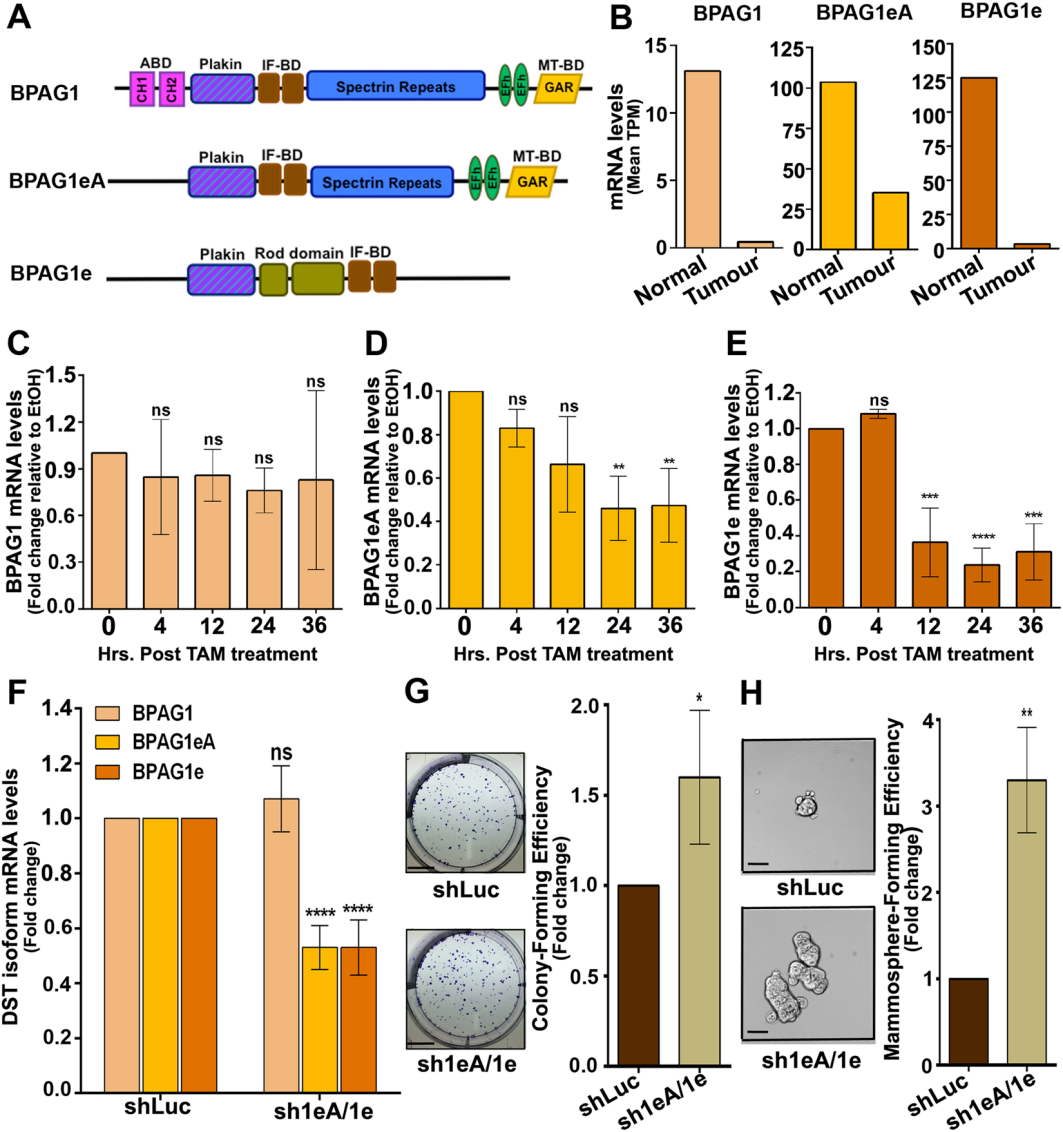
BPAG1eA and BPAG1e are downregulated by Src activation and in breast tumours and promote transformation of MCF10A cells when knocked down. **(A)** Schematic domain representation of the human BPAG1 long isoforms and the two shorter BPAG1eA and BPAG1e isoforms. **(B)** Median number of Transcripts Per Million (Median TPM) for BPAG1, BPAG1eA and BPAG1e in 114 normal breast tissues or 1097 breast tumour samples. **(C–E)** Ratio of BPAG1 **(C)** or BPAG1eA **(D)** or BPAG1e **(E)** mRNA levels between TAM- and EtOH-treated MCF10A-ER-Src cells for the same time points (0, 4, 12, 24 and 36 hours), normalized to GAPDH. Data are from three biological replicates, performed in triplicates. **(F)** Fold change of BPAG1 or BPAG1eA or BPAG1e mRNA levels between shLuc- and shBPAG1eA/1e-expressing MCF10A cells, normalized to GAPDH. Data are from three biological replicates performed in triplicates. **(G)** (Left panels) Representative images of colonies from shLuc- or shBPAG1eA/1e-expressing MCF10A cells grown in clonogenic assays. Scale bars represent 5 mm. (Right panel) Fold change in colony-forming efficiency between shLuc- and shBPAG1eA/1e-expressing MCF10A cells. Data are from three biological replicates performed in triplicates. **(H)** (Left panels) Representative images of shLuc- or shBPAG1eA/1e-expressing MCF10A mammospheres. Scale bars represent 50 μm. (Right panel) Fold change in mammosphere-forming efficiency between shLuc- and shBPAG1eA/1e-expressing MCF10A cells. Data are from three biological replicates performed in triplicates. For all quantifications, error bars indicate SD.; ns indicates non-significant; *indicates P < 0.05; **indicates P < 0.005; ***indicates P < 0.001; ****indicates P < 0.0001.

To test the possibility that the tumour suppressor effect of DST is endorsed by specific DST spliced variants, we searched for the DST isoform downregulated in breast tumour samples. Alternative splicing of the DST gene results in 35 splice variants. However, only four and five complete annotated transcripts are listed in the Ensembl genome browser (Release 87) and NCBI gene database, respectively (Supplementary Table S4), with isoforms 2 and 3 from the NCBI gene database being pooled as identical transcripts by Ensembl (isoform DST-207). We therefore only consider DST-207, DST-202, DST-201 and DST-205. DST-207 and DST-202 contain the two CH, the plakin, the IF-BD, the EF-hand, the GAR domains and the spectrin-repeat rod (referred as BPAG1, Fig. 7A). In contrast, DST-201 contains the Plakin, the spectrin, the EF-hand and the GAR domains (referred to as BPAG1eA, Fig. 7A), while DST-205 encompasses the Plakin, the rod and the IF-BD domains (referred as BPAG1e, Fig. 7A). Searching through the ISOexpresso database (http://wiki.tgilab.org/ISOexpresso/)^48^, we found that BPAG1, BPAG1eA and BPAG1e were all expressed in normal breast tissues and were reduced by 29, 2.9 and 37 folds, respectively, in breast tumour samples (Fig. 7B). We next examined if all DST isoforms were downregulated during transformation of the TAM-treated MCF10A-ER-Src cell line (Fig. 1) using pairs of primers that specifically amplify BPAG1, BPAG1eA or BPAG1e. In contrast to the long BPAG1 isoforms, whose expression was not significantly altered during the 36 hours of TAM treatment (Fig. 7C), BPAG1eA and BPAG1e mRNA levels were reduced by 69% and 53%, starting 24 and 12 hours respectively after TAM treatment (Fig. 7D,E). These observations suggest that BPAG1eA and BPAG1e could function as the predominant DST tumour suppressor variants in breast epithelial cells.

### BPAG1e and/or BPAG1eA prevent transformation in MCF10A cells

To test if the downregulation of BPAG1e and BPAG1eA is sufficient to promote cellular transformation, we generated stable MCF10A cells carrying Tet-inducible shRNA against the BPAG1eA and BPAG1e isoforms (sh1eA/1e). Unlike shDST-expressing cells, which showed a reduction of all DST isoforms in both MCF10A-ER-Src and MCF10A cells (Supplementary Fig. S3), treating sh1eA/1e cells with Tet had no effect on BPAG1 mRNA levels, but reduced BPAG1eA and BPAG1e mRNA levels by 47% (Fig. 7F). Knocking down BPAG1eA and BPAG1e was sufficient to potentiate the colony-forming ability of MCF10A cells in clonogenic assays (Fig. 7G), as well as their mammosphere-forming capacity (Fig. 7H). Consistent with a role of BPAG1eA and/or BPAG1e as tumour suppressors in breast cells, MCF10A cells knocked down for BPAG1eA and BPAG1e using independent shRNA, displayed reduced BPAG1eA and BPAG1e mRNA expression specifically and formed higher number of mammospheres than control shLuc cells (Supplementary Fig. S3). Thus, BPAG1e and/or BPAG1eA prevent the acquisition of transformed features in MCF10A cells and could endorse most of DST tumour suppressive activity.

## Discussion

### Control of Hippo signalling by DST

Our observations support a role of YAP downstream of the loss of DST in promoting the transformation of MCF10A cells. DST-depleted cells show increased YAP nuclear localization and expression of YAP target genes, and their growth is suppressed when treated with an inhibitor of YAP activity. Furthermore, *in vivo*, the *Drosophila* DST Shot restricts tissue growth by limiting Yki activity. DST could feed into one of the numerous inputs regulating YAP^49^. Among these, stiff substrate-induced cell spreading, which involves mechanical signals controlled by the actin cytoskeleton, triggers YAP activity. Conversely, cell rounding and reduced adhesive area generated by softer substrates, cause cytoplasmic retention and YAP inhibition^22,23,50,51^. DST-depleted MCF10A cells, alike keratinocytes knocked down for BPAG1e^52^, exhibit reduced cell spreading and cell-substratum adhesion and fail to localize Paxillin and Vinculin at FAs. Thus, increased YAP activity in these cells unlikely results from higher cell-substrate tension. Still, DST could impact on YAP activity through the control of the actin cytoskeleton. This effect unlikely involves a direct control of F-actin by DST, as BPAG1e and BPAG1eA, the two DST variants that prevent transformation in MCF10A cells, do not contain the F-actin-binding calponin-homology domains. Instead, DST could affect F-actin by controlling actin-binding proteins. In agreement with this possibility, the activity of the actin-severing factor Cofilin is reduced in BPAG1e-deficient keratinocytes^52,53^. As Cofilin restrains YAP activity in human cells and in *Drosophila* epithelia^22,54^, DST could limit YAP activity through Cofilin regulation. DST could also restrict YAP activity by keeping in check the actin-associated LIM domain protein Zyxin. We show that DST prevents Zyxin accumulation in MCF10A cells. In TAM-treated MCF10A-ER-Src, the downregulation of DST is also associated with the upregulation of Zyxin^34^. On possibility is that DST tethers Zyxin at FAs through maintaining FA integrity. In DST-depleted cells, Zyxin mislocalization could boost YAP activity, either through F-actin regulation, reminiscent to one of Zyxin’s functions in *Drosophila* epithelia^26^ and/or by stabilizing LATS^27,28^. According to the latter, DST-depleted MCF10A cells show reduced LATS protein levels. In support of a causal relationship between defective cell–substrate adhesion and impaired Hippo signalling, fibroblasts lacking the α-tubulin K40 acetyltransferase (αTat1), also display reduced number of FAs, impaired cell-substrate adhesion and fail to activate Hippo signalling upon cell-cell contacts^55^. Higher YAP activity due to the loss of DST could, in turn, initiate a positive feedback loop, which sustains its activity, as inhibiting YAP function in the triple-negative breast cancer cell line CAL51 upregulates DST and reduces Zyxin accumulation^51^.

### A role for the BPAG1eA and/or BPAG1e DST isoforms in tumour suppression

Our observations suggest that the BPAG1eA and/or BPAG1e isoforms endorse the tumour suppressor function of DST. Consistent with this hypothesis, reducing all DST variants (shDST#2) or only BPAG1eA and BPAG1e by around 50%, potentiates to similar extends the colony- and mammosphere-forming abilities of MCF10A cells. Moreover, Src-induced transformation in MCF10A cells is associated with the downregulation of BPAG1eA and BPAG1e, but not with that of the long BPAG1 isoforms. How could BPAG1eA/BPAG1e impact on YAP activity? BPAG1eA contains a GAR microtubule-binding domain. Tao-1, known for destabilizing microtubules, controls Hippo pathway activity^56,57^. Moreover, acetylated microtubules by αTat1 have been proposed to regulate contact inhibition of proliferation through the Hippo pathway^55^. Thus, through its microtubule-binding activity, BPAG1eA could support FA and F-actin integrity, therein keeping in check YAP. Alternatively, the keratin-anchoring function of BPAG1e to the cell surface at the site of hemidesmosomes^58–63^ could hold strong cell-matrix adhesions through FAs assembly and F-actin integrity. In agreement with this model, breast epithelial and myoepithelial cells assemble hemidesmosomes^64^. Moreover, hemidesmosomes can maintain the size of FAs^65^. Furthermore, carcinoma *in situ* and invasive breast cancer cells lack hemidesmosomes^64^. However, we cannot exclude that the long BPAG1 isoforms also exhibit tumour suppressor effects in breast epithelial cells, as all DST isoforms are downregulated in breast tumour samples. Moreover, knocking down all DST isoforms in EtOH-treated MCF10A-ER-Src cells has stronger effect on growth than TAM-treated MCF10A-ER-Src cells expressing shLuc.

### DST and cancer progression

Diverse observations suggest that the loss of DST could promote breast cancer progression irrespectively of the hormonal status. We show that DST has tumour suppressor activity in breast epithelial cells. Accordingly, DST has also been shown to prevent tumour growth and invasion in a MCF10ADCIS.com xenograft model^35^. Moreover, DST is downregulated in ER-positive DCIS and IDC and in ER-negative IDC. MCF10A cells with conditional Src induction also downregulate BPAG1eA and BPAG1e^33,34^. In addition, reducing further DST levels in these cells potentiates cell growth. The downregulation of DST in this inducible cell line could potentiate YAP activity, as the set of genes affected in TAM-treated MCF10A-ER-Src cells^34^ show a significantly 5.31 folds enrichment (36/226 genes, p < 0.0001, Hypergeometric test) for YAP target genes in MCF10A cells^66^. In this model, which recapitulates the multistep process of Src-induced cellular transformation, cells transiently assemble stress fibres and FAs, leading to sustained cell proliferation 12 hours after induction. This state does not appear to involve YAP activation but depends on ERK and take place concomitantly to the downregulation of DST^33^. As reducing DST function in MCF10A cells affect FA integrity, the downregulation of DST in TAM-treated MCF10A-ER-Src cells could allow for FA and stress fibres disassembly after 12 hours of TAM treatment, consequently impeding Hippo pathway activity, and the progression towards a fully transformed state. Consistent with a role of Hippo signalling in breast cancer, LATS1 and LATS2 are downregulated in breast cancer and their depletion results in the acquisition of cancer-like features^67^. In contrast, high levels of Zyxin and nuclear YAP were reported in breast cancer tissues and both causes the acquisition of transformed features in breast cells^28,68–70^. BPAG1e is also downregulated in nasopharyngeal carcinoma^71^. However, in invasive squamous cell carcinoma, BPAG1e is upregulated and confers cell migration, invasion and tumorigenic potential to oral squamous cell carcinoma cells^36,37^. Thus, in cancer cells, DST function could be context dependent. Consistent with this possibility, in the fly, while Shot restricts the overgrowth of wild type epithelia, it promotes growth in tissues overexpressing *wts* or knocked down for *yki*. Importantly, Zyxin also displays tissue-dependent opposite effects on cancer progression^72^. Thus, the tumour suppressor versus oncogenic roles of BPAG1e could rely upon Zyxin functions and interacting partners in distinct epithelia. However, we cannot exclude that distinct DST isoforms have opposite effects in cancer progression.

Taken together, our data demonstrate that the loss of DST in MCF10A cells triggers the acquisition of transformed features and potentiates YAP activity. Our observations are consistent with a model by which the tumour suppressor function of DST could be restricted to the shorter DST isoforms BPAG1eA and/or BPAG1e. As both of these isoforms are downregulated by Src and in breast tumour samples, our work places BPAG1eA and/or BPAG1e as essential breast cancer suppressors. Deeper characterization of each DST isoform would shed light on their role in carcinogenesis and in Hippo signalling activity.

## Materials and Methods

### Cell lines and culture conditions

MCF10A pBABE-puro cells and MCF10A-ER-Src cells were kindly provided by K. Struhl^38^. HEK293T cells were a kindgift from M.J. Amorim lab at the Instituto Gulbenkian de Ciência, Portugal. MCF10A pBABE-puro, MCF10A-ER-Src cells as well as their derivatives MCF10A-shLuc, MCF10A-shDST, MCF10A-shDST#2, MCF10A-shBPAG1eA/1e, MCF10A-shBPAG1eA/1e#2, MCF10A-ER-Src-shLuc and MCF10A-ER-Src-shDST were cultured in 5% CO_2_, humidified atmosphere at 37 °C in DMEM/F12 media (Thermo Fisher Scientific, Waltham, MA, USA; 11039–047) supplemented with 5% charcoal stripped horse serum (Gibco/Thermo Fisher Scientific, Waltham, MA, USA; 16050–122), 20 ng/ml EGF (Peprotech, London, UK; AF-100-15), 100 ng/ml Cholera Toxin (Sigma-Aldrich, Darmstadt, Germany; C-8052), 0.5 µg/ml Hydrocortisone (Sigma-Aldrich; H0888), 10 µg/ml Insulin (Sigma-Aldrich; I9278), 0.5 µg/ml Puromycin (Calbiochem, Darmstadt, Germany; 540411), and 1X Penicillin-Streptomycin (Thermo Fisher Scientific; 15070063). All cell lines were tested regularly for mycoplasma contamination and tested negative. After thawing, cell lines stably transfected with shLuc, shDST or shBPAG1eA/1e were cultured for two passages in media supplemented with 900 µg/ml of G418 (Thermo Fisher Scientific; 11811031).

HEK 293 T cells were grown in DMEM media (Thermo Fisher Scientific; 31053028) supplemented with 10% Tetracycline-free FBS (Clontech, France; 621105), 2 mM L-glutamine (Thermo Fisher Scientific; 25030024), 1 mM sodium pyruvate (Thermo Fisher Scientific; 11360070) and 1X Penicillin-Streptomycin.

### Stable cell line generation

pLKO-TET-Neo vector containing shLuc, shDST or shBPAG1eA/1e were generated as described in Wiederschain *et al*.^73^. shRNA seed sequences were designed using Dharmacon siDesign Center (Supplementary Table S1). The pLKO-TET-Neo vector (Addgene, Watertown, MA, USA; Plasmid # 21916) was digested with ECoRI and AgeI, ligated with shRNA oligos and transformed into E.coli DH5alpha strain. Plasmids were purified using the WizardPlus Midiprep Kit (Promega, Madison, WI, USA; A7640) and transfected into HEK 293 T cells along with envelope and packaging plasmids using calcium phosphate transfection. Viral supernatants were collected 24 and 48 hours later, pooled together and used to infect MCF10A-pBABE-puro and MCF10A-ER-Src cells grown in media containing 8 µg/ml polybrene (Merck Millipore, Darmstadt, Germany; TR-1003-6) on two consecutive days. 48 hours post infection, cells were split and grown in media containing 900 µg/ml of G418 (Thermo Fisher Scientific, Waltham, MA, USA; 11811031) for selection. The levels of the housekeeping genes GAPDH, Actin and Histone H3 were compared by western blot between established stable cell lines and original cell lines to select for clones used in this study.

### Drug treatments

To determine the expression of DST in MCF10A-ER-Src cells, cells were seeded to reach 30% confluency 24 hours post-seeding and induced with 1 µM 4OH-TAM (Sigma-Aldrich, Darmstadt, Germany; H7904) or equal volume of EtOH in complete growth medium for the indicated time. To analyse the effect of reducing further DST in MCF10A-ER-Src-shLuc and MCF10A-ER-Src-shDST cells, cells were treated with 400 ng/ml Tet (Sigma-Aldrich; T7660) dissolved in 70% EtOH for 36 hours, 24 hours post-seeding, and treated with media containing 1 µM 4OH-TAM (Sigma-Aldrich; H7904) or an equal volume of EtOH for an additional 36 hours. To quantify the growth of MCF10A-ER-Src cells expressing shLuc or shDST, 3 000 cells per well in triplicates were seeded in 96 well plates for 24 hours and treated with Tet and TAM or EtOH as previously described. To analyse the effects of knocking down DST or BPAG1eA/1e in MCF10A cells, MCF10A-shLuc, MCF10A-shDST and MCF10A-shBPAG1eA/1e cells were seeded to reach 20% confluency after 24 hours and treated with 400 ng/ml Tet in complete growth media for 72 hours. For testing the effect of knocking down DST on YAP sub-cellular localization and expression of its target genes, the number of MCF10A-shLuc and MCF10A-shDST cells seeded was determined to reach comparable percentage of cell confluency 72 hours after Tet treatment (Supplementary Table S2). Cells were serum starved 12–14 hours before ending each experiment. For co-treatment with Tet and Verteporfin (VP), 40 000 cells per well were seeded in 24 well plates. After 24 hours, cells were pre-treated with 7.5 µM VP (Sigma-Aldrich; SML0534–5MG) or equivalent volume of DMSO in complete growth medium for 4 hours. Media was then replaced with media containing Tet and DMSO or VP for 24, 48 or 72 hours. For all experimental settings, media was changed every 24 hours.

MCF10A-shDST replicate treated with Tet for 72 hours and used to evaluate the expression of the YAP target genes CTGF, CYR61 and ITGB6 in Fig. 4B.

### Real-time PCR

Total RNA was isolated using the RNAeasy Kit (Qiagen, Hilden, Germany; 74104). 0.5 µg of purified RNA samples were used for first strand cDNA synthesis using random hexamer primers (Supplementary Table S3), according to manufacturer’s instructions (Roche Molecular Systems Inc., Pleasanton, CA, USA; Ref # 04896866001). Real time quantitative PCR were performed in triplicates in 384 well plates using the iTaq Universal SyBR Green Supermix (Bio-Rad, Hercules, CA, USA; 1725125) in either the QuantStudio 7 Flex Real -Time PCR Instrument (Applied Biosystems, Foster City, CA, USA) or the CFX384 Touch Real- Time PCR Detection System (Bio-Rad). Relative fold changes were calculated using the ΔΔCt method^74^.

### Identification of DST isoforms

DST/BPAG1 sequences were retrieved from the Ensembl database and NCBI. Primer-BLAST was used for designing isoform sequence primers (Supplementary Table S3). Current nomenclature for annotated isoforms and accession numbers are listed in Supplementary Table S4.

### Fly Strains and Genetics

Fly stocks used were *nub-*Gal4^75^, *hh-*Gal4, agift from T. Tabata (University of Tokyo, Japan); *tub-*Gal80^ts^; UAS-*Myc::wts*^76^; UAS-*L(A)-GFP*^77^, UAS-*shot-RNAi*^*GL01286*^, *shg*^*k03401*^^78^ and UAS-*yki-RNAi*^*4005R-2*^ (NIG-Fly). To analyse the effect of knocking down *shot* on the expression of Yki target genes, *tub-*Gal80^ts^; UAS-*shot*-RNAi^GL01286^ females were crosses with *hh-*Gal4, UAS-*GFP* males. Adults were left to lay eggs for 48 hours. Progeny were maintained at 18 °C for 6 days and switch to 25 °C until the end of third instar larvae. All other crosses were maintained at 25 °C. Larvae were dissected at the end of third instar.

### Clonogenic assay

500 MCF10A-shLuc or MCF10A-shDST or MCF10A-shDST#2 or MCF10-shBPAG1eA/1e cells treated with Tet for 72 hours were seeded per well in triplicates in 6 well plates and incubated for 9 days in complete growth media containing Tet. Media was replaced every 3 days. Colonies were then fixed with 3.5% paraformaldehyde and stained with 0.005% crystal violet dissolved in 20% ethanol solution. Colonies were counted manually. Colony forming efficiency (C.F.E.) was calculated as – (Number of colonies/Number of cells seeded) *100. Fold changes in C.F.E. were determined by normalizing to the C.F.E. of MCF10A-shLuc cells.

### Drug resistance assay

To treat MCF10A-shLuc and MCF10A-shDST cells with Tet and Doxorubicin, cells were seeded to reach 20% confluency at 24 hours post-seeding and then treated with 400 ng/ml Tet (Sigma-Aldrich; T7660). After 48 hours, media containing 400 ng/ml Tet and 250 nM of Doxorubicin (Sigma-Aldrich; D1515) dissolved in PBS was applied for an additional 24 hours. 1 000 cells per well were then seeded in 6 well plates in triplicates and incubated for 9 days. Medium was replaced every 3 days with fresh media containing Tet. At the end of 9 days, colonies were fixed with 3.5% paraformaldehyde and stained with 0.005% crystal violet diluted in 20% ethanol solution. Colonies were counted manually. Percent surviving fraction was calculated as – (Number of colonies formed/Number of cells seeded*Plating Efficiency)^79^.

### Soft agar assay

5 000 MCF10A-shLuc or MCF10A-shDST cells treated with Tet for 72 hours were resuspended in complete growth medium containing 0.3% gelling agarose (Seakem LE Agarose; Lonza, Switzerland, # 50004) and plated in triplicates in 6 well plates on the top of a solidified layer of 0.5% agarose in 500 µl complete growth media containing Tet. Growth medium containing Tet was replaced every 3 days. After 3 to 4 weeks, plates were stained with 0.005% crystal violet dissolved in 20% ethanol. Number of colonies was counted using the Analyse Particles tool In Fiji. Only colonies greater than 50 µm in diameter were scored as positive. Colony forming efficiency (C.F.E.) was calculated as – (Number of colonies/Number of cells seeded) *100. Fold changes in C.F.E. were determined by normalizing to the C.F.E. of MCF10A-shLuc cells.

### Mammosphere formation assay

7 500 MCF10A-shLuc or MCF10A-shDST or MCF10A-shDST#2 or MCF10-shBPAG1eA/1e or MCF10-shBPAG1eA/1e#2 cells treated with Tet for 72 hours were seeded per well in triplicate in 6 well plates coated with Poly-HEMA (Sigma-Aldrich; P3932). Cells were grown in DMEM/F12 media supplemented with 1X B27 supplement (Thermo Fisher Scientific; 17504001), 40 µg/ml insulin, 0.5 µg/ml hydrocortisone, 20 ng/ml EGF and 1X Penicillin-Streptomycin and 400 ng/ml Tet. Plates were incubated undisturbed for 6 days to allow mammosphere growth. Images were acquired on a commercial Leica High Content Screening microscope Leica DMI6000 equipped with a Hamamatsu Flash Orca 4.0 sCMOS camera (Leica Microsystems, Germany), using a 10 × 0.30 NA objective. Number of mammospheres with diameter greater than 50 µm was counted by using the Analyse Particles function of Fiji. Mammosphere forming efficiency (M.F.E.) was calculated as – (Number of mammospheres/Number of cells seeded) *100. Fold changes in M.F.E. were determined by normalizing to the M.F.E. of MCF10A-shLuc cells.

### Cell growth assay

The Cell Counting Kit-8 kit from Dojindo, Japan (277CK04–11) was used for determining relative cell number according to manufacturer’s instructions. Cells were incubated for 2 hours with the CCK-8 reagent and the absorbance (optical density, O.D.) at 450 nm was detected using a Synergy Mx (BioTek, Vermont, USA) microplate reader. Absorbance was normalized to absorbance before treatment.

### Luciferase reporter assay

15 × 10^4^ MCF10-shDST cells per well were seeded in 6 well plates, allowed to adhere for 24 hours and further grown for 24 hours in the presence or absence of Tet. Cells were then transiently transfected with 30 ng of pRL-TK plasmid and 720 ng of either pGL3–49 or pGL3–4XGTIIC-49^44^ plasmid (A kindgift from Nic Tapon, The Francis Crick Institute) using Lipofectamine 2000 (Thermo Fisher Scientific; 11668019). Cells were then allowed to grow for 48 hours in complete growth media in the presence or absence of Tet, trypsinized and analyzed using the Dual Luciferase Reporter Kit (Promega, Madison, WI, USA; E1910) as per manufacturer’s instructions. Luminescence was measured in triplicates for each sample on a Synergy Mx (BioTek) microplate reader. Relative luciferase activity was quantified by normalizing Firefly luciferase activity to their respective Renilla luciferase activity.

### Immunoblotting analysis and quantifications

For protein extraction, cells were trypsinized and pellet resuspended in lysis buffer (50 mM Tris-HCl - pH 7.5, 150 mM NaCl, 1 mM EDTA, 1 mM EGTA, 1% Triton X-100, 1X Protease and Phosphatase inhibitors) for 20 minutes. Supernatants were collected after centrifugation at 14 000 rpm at 4 °C for 30 minutes. Protein concentration was estimated using Protein Assay Dye Reagent (Bio-Rad, Hercules, CA, USA; 5000006). 5X Laemelli buffer was then added to cells lysates before boiling for 5 minutes. 40 µg of protein extracts were then loaded for each sample, resolved by SDS-PAGE electrophoresis and transferred to PVDF membranes (GE Healthcare, Chicago, IL, USA; 10600023). Membranes were blocked with 3% BSA in TBS 0.1% Tween-20 and incubated with anti-GAPDH (1:5 000; Sigma-Aldrich; G9545) as loading control, anti-Zyxin (1:1000; Sigma-Aldrich; HPA004835), anti-LATS1/2 (1:1 000; Abcam, Cambridge, UK; ab70565) anti-activated Src (1:1000; Invitrogen, 44–660G). Detection was performed using HRP-conjugated antisera (1:2 500–1:5 000; Jackson ImmunoResearch, Cambridgeshire, UK; JIR-711-035-152) and chemiluminescent detection using Immobilon Western Chemiluminescent HRP Substrate (Merck Millipore, Darmstadt, Germany; WBKLS0100) on a Bio-Rad ChemiDoc XRS + system (Bio-Rad). Intensity of bands was measured using the Bio-Rad Image Lab software (Bio-rad). To quantify relative fold change in protein levels, band intensities of protein of interest were first normalized to band intensity of respective loading control band and the normalized intensity of MCF10A-shDST was divided by that of MCF10A-shLuc.

### Immunofluorescence analysis and quantifications

For focal adhesion staining of MCF10A-shLuc and MCF10A-shDST, cells treated with Tet for 72 hours were trypzinized, reseeded on poly-L-lysine (Sigma-Aldrich; P8920) coated coverslips, allowed to attach and further grown for 48 hours in media containing Tet. For determining YAP sub-cellular localization, MCF10A-shLuc and MCF10A-shDST cells were seeded on poly-L-lysine coated coverslips, grown in complete growth media containing Tet for 72 hours and cells were serum starved for the last 12–14 hours before the end of each experiment. Cells were then fixed with 4% Formaldehyde solution in PBS at pH 7 for 10 minutes, permeabilized with 0.1% Triton X-100 and blocked with cytoskeletal binding buffer [10 mM MES, 150 mM NaCl, 5 mM EGTA, 5 mM MgCl_2_, 5 mM glucose - pH 6.1, containing 2% (v/v) FBS and 1% (w/v) BSA]. Primary antibodies were incubated overnight at 4 °C in blocking solution. Coverslips were then washed twice with PBS and incubated with secondary antibodies and with Rhodamine-conjugated Phalloidin (Sigma-Aldrich; P1951) at 0.3 mM in blocking solution for 1 hour at RT. For DAPI staining, cells were washed thrice in PBS and stained with 2 µg/ml DAPI (Sigma-Aldrich; D1377) for 5 min in PBS, washed again with PBS and mounted in Vectashield (Vector Laboratories, Burlingame, CA, USA; H-1000). Primary antibodies used were Anti-Paxillin (1:500; BD Biosciences, San Jose, CA, USA; BD #610051), Anti- Zyxin (1:25; Sigma-Aldrich; HPA004835), anti-vinculin (1:100; Sigma-Aldrich; hVIN-1 #V9131), anti-YAP (1:200; Santa Cruz Biotechnology, Inc; Heidelberg, Germany; sc-101199), anti-Lamin A/C (1:500; Abcam; ab108595). Secondary antibodies were obtained from Jackson ImmunoResearch and used at 1:200 dilution. Confocal Z-series stacks were acquired on a Leica SP5 confocal, using a 63 × 1.3NA Oil immersion objective (Leica Microsystems).

Blind quantifications of the percentages of cells with predominant nuclear YAP were performed separately by P. S. Guerreiro and F. Janody on 3 biological replicates by manual counting.

For wing imaginal discs of third instar larvae, staining were performed by dissecting larvae in phosphate buffer at pH 7 (0.1 M Na_2_HPO_4_, 0.1 M NaH_2_PO_4_ at a 72:28 ratio). For analysing the effects of overexpressing *wts* or knocking down *yki* on the growth of *nub* > *shot-RNAi*-expressing wing discs, only female larvae were dissected. Samples were then fixed in 4% formaldehyde in PEM (0.1 M PIPES pH 7.0, 2 mM MgSO_4_, 1 mM EGTA) for 20 to 30 min, rinsed in phosphate buffer 0.2% Triton for 15 min and incubated with mouse anti-β-Galactosidase (1:200; Promega; Z37832000) or rabbit anti-Expanded (1/200; agift from A. Laughon, University of Wisconsin, Madison, WI, USA) or mouse anti-Discs large (1:50; Developmental Studies Hybridoma Bank, Iowa, USA, 4F3) overnight at 4 °C. Discs were then rinsed 3 times 10 min in phosphate buffer 0.2% triton, incubated for 1 h with TRITC- or Cy5-conjugated Donkey anti-mouse or anti-rabbit (Jackson ImmunoResearch) in phosphate buffer 0.2% triton supplemented with 10% horse serum. Samples were then rinsed 3 more times 10 min before being mounted in Vectashield. Fluorescence images were obtained on a Leica SP5 Live confocal microscope using a 10X (zoom twice) or 40X water objectives. The NIH Image J program was used to quantify wing disc area. Each disc was outlined and measured using the *Area* function, which evaluates size in square pixels. To quantify the ratio of the *nub* > *GFP* domain over the total wing disc area, the ratio between the area of the GFP domain and the area of the whole disc domain, measured using the *Area* function for each disc, were performed.

### Cell-Substratum Adhesion Assay

1.6 × 10^5^ MCF10A-shLuc and MCF10A-shDST cells per well were seeded in 24 well plates in triplicates and incubated either in complete growth media or DMEM/F12 media without supplements for 16 hours. After removing media, wells were rinsed with ice-cold PBS^++^ buffer (PBS containing 1 mM CaCl_2_ and 1 mM MgCl_2_), fixed with ice-cold methanol and incubated in 0.5% crystal violet dissolved in 20% ethanol for 20 minutes at room temperature. Excess crystal violet was discarded, cells were washed with PBS and incubated with methanol at room temperature to recover crystal violet from adherent cells. Absorbance at 590 nm was detected using a Synergy Mx (BioTek) microplate reader. Percentage of adhesion was calculated by considering adhesion in complete growth media as 100% as described in^80^.

### Cell Spreading Assay

For measuring cell spreading, 5 000 MCF10A-shLuc and MCF10A-shDST cells were seeded per well in 24 well plates. Phase-contrast images were taken 6 hours post-seeding at 10X magnification. Cell area was determined using the Fiji software by detecting cell boundaries with the Find Edges function and acquiring cell area measurements with the Analyze Particles function.

### Statistical analysis

The Prism 7 (GraphPad Inc., San Diego, CA, USA) software was used for all statistical analysis. Unpaired t-test was used to compare two groups/treatments except for comparison of final cell number used to determine YAP target gene expression, where a paired t-test was used. For comparing alteration in total DST/BPAG1, BPAG1eA and BPAG1e mRNA levels in TAM-treated MCF10A-ER-Src cells, growth rates and distal wing disc area, multiple comparisons using one-way ANOVA were performed. p-values < 0.05 were considered to be significant. No statistical test was used to determine the number of biological replicates to be performed.

## Supplementary information

Supplementary Information

## Data Availability

All data and cell lines generated in this work are available upon request.
