# Supplementary material for: The spectraplakin Dystonin antagonizes YAP activity and suppresses tumourigenesis
Source: Sci Rep. 2019 Dec 27;9:19843. doi: 10.1038/s41598-019-56296-z (PMC6934804; doi:10.1038/s41598-019-56296-z)
Supplement: Supplementary file 1 — Supplementary Information [file 41598_2019_56296_MOESM1_ESM.pdf]

# The spectraplakin Dystonin antagonizes YAP activity and suppresses tumourigenesis

**Praachi B. Jain, Patrícia S. Guerreiro, Sara Canato and Florence Janody**

**Supplementary Information**

Supplementary Figure S1: Page 2
Supplementary Figure S2: Page 3
Supplementary Figure S3: Page 4-5
Supplementary Table S1: Page 6
Supplementary Table S2: Page 6
Supplementary Table S3: Page 7
Supplementary Table S4: Page 7

## Supplementary Figures

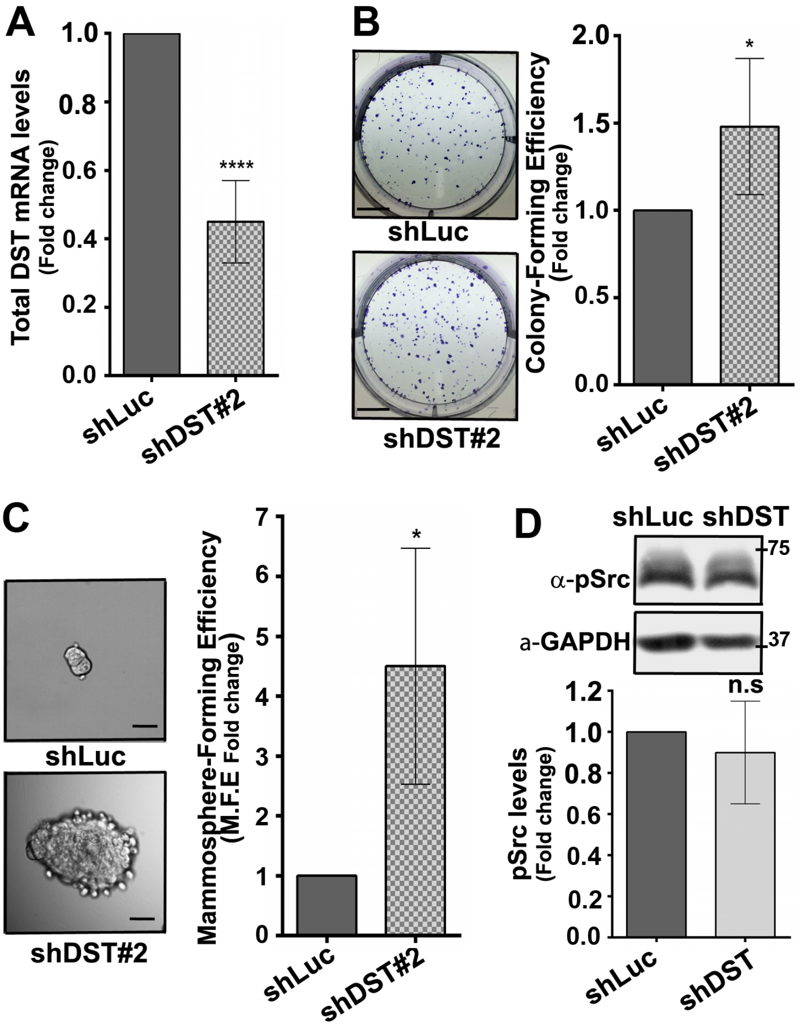


**Supplementary Figure S1: Knocking down DST promotes transformation in MCF10A cells independently of Src activation. (A)** Fold change of total DST mRNA levels between shLuc- and shDST#2-expressing MCF10A cells, normalized to GAPDH. Data are from four biological replicates performed in triplicates. **(B)** (Left panels) Representative images of colonies from shLuc- or shDST#2-expressing MCF10A cells grown in clonogenic assays. Scale bars represent 5 mm. (Right panel) Fold change in colony-forming efficiency between shLuc- and shDST#2-expressing MCF10A cells. Data are from four biological replicates performed in triplicates. **(C)** (Left panels) Representative images of shLuc- or shDST#2-expressing MCF10A mammospheres. Scale bars represent 50 μm. (Right panel) Fold change in mammosphere-forming efficiency between shLuc- and shDST#2-expressing MCF10A cells. Data are from three biological replicates performed in triplicates. **(D)** (Upper panels) Western blots on protein extracts from shLuc- or shDST-expressing MCF10A cells, blotted with anti-pSrc (upper bands) or anti-GAPDH (lower bands). (Lower panels) Ratio of pSrc levels between shLuc- and shDST-expressing MCF10A cells, normalized to GAPDH. Data are from three biological replicates. For all quantifications, error bars indicate SD.; ns indicates non-significant; * indicates $P<0.05$; **** indicates $P<0.0001$.

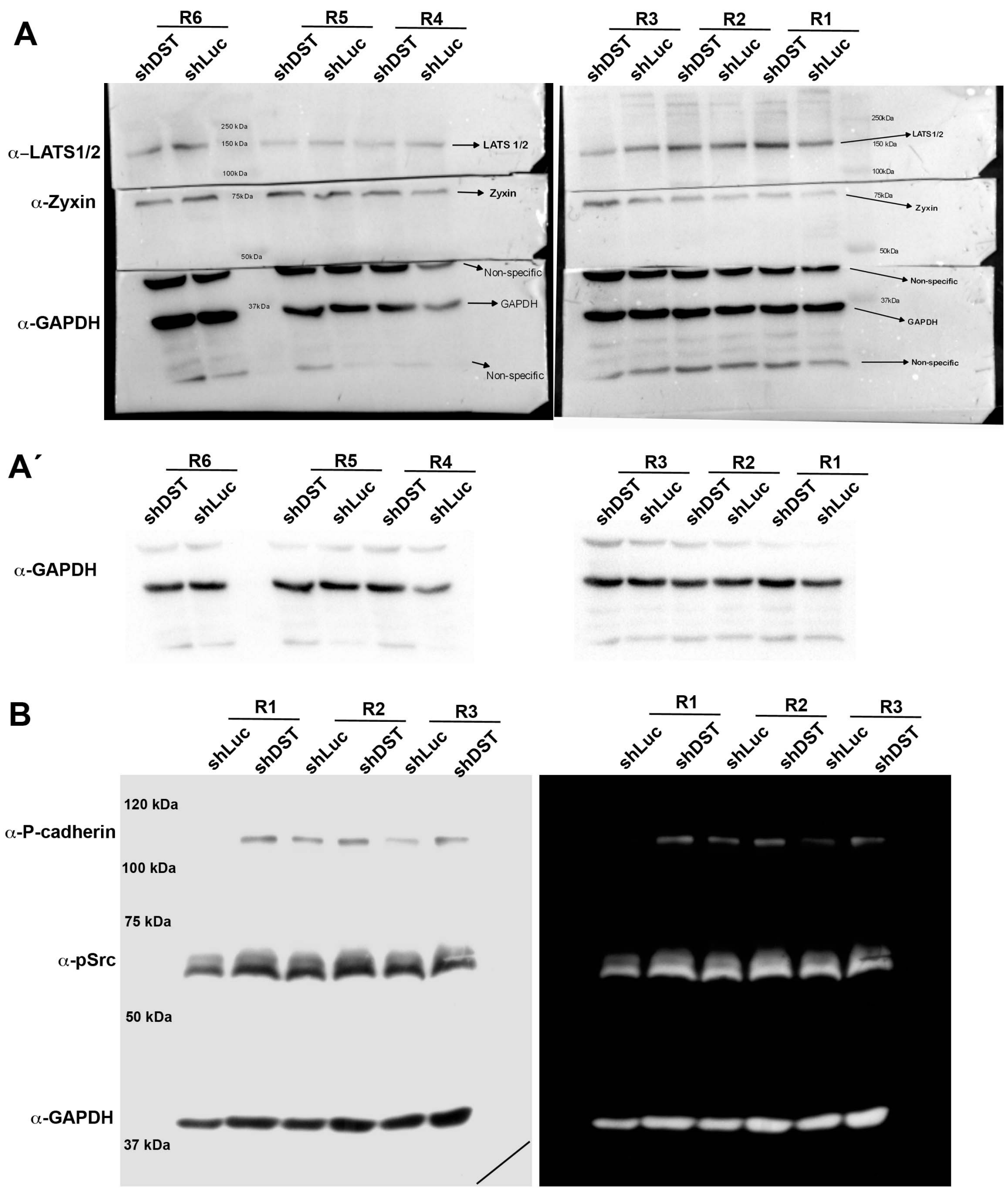


**Supplementary Figure S2: Knocking down DST promotes Zyxin accumulation, destabilizes LATS1/2, but has no major effect on pSrc levels. (A)** Western blots on protein extracts from shLuc- or shDST-expressing MCF10A cells, blotted with anti-LATS1/2 (upper blots), or anti-Zyxin (middle blots) or anti-GAPDH (lower blots). R1, R2, R3, R4, R5 and R6 represent six biological replicates. Following protein transfer, membranes were cut in three pieces according to the molecular weight of the ladders and used for blotting with the anti-LATS1/2 (upper blots) or anti-Zyxin (middle blots) or anti-GAPDH (lower blots) antibodies. Blots loaded with replicates R1 to R3 (right) were exposed for 25 seconds. Blots loaded with replicates R4 to R6 (left) were exposed for 60 seconds. **(A′)** corresponds to lower exposure of membranes blotted with the anti-GAPDH antibody shown in A. The blot loaded with replicates R1 to R3 (right) was exposed for 0.82 seconds. The blot loaded with replicates R4 to R6 (left) was exposed for 21 seconds. **(B)** Western blots on protein extracts from shLuc- or shDST-expressing MCF10A cells, blotted with the anti-P-cadherin (upper bands) or anti-pSrc (middle bands) or anti-GAPDH (lower bands) antibodies. R1, R2 and R3 represent three biological replicates loaded on the same gel. The original film is shown on the right panel. The black line on the right bottom corner marks the reference for loading. Quantification of each biological replicate was performed using the blot shown on the left.

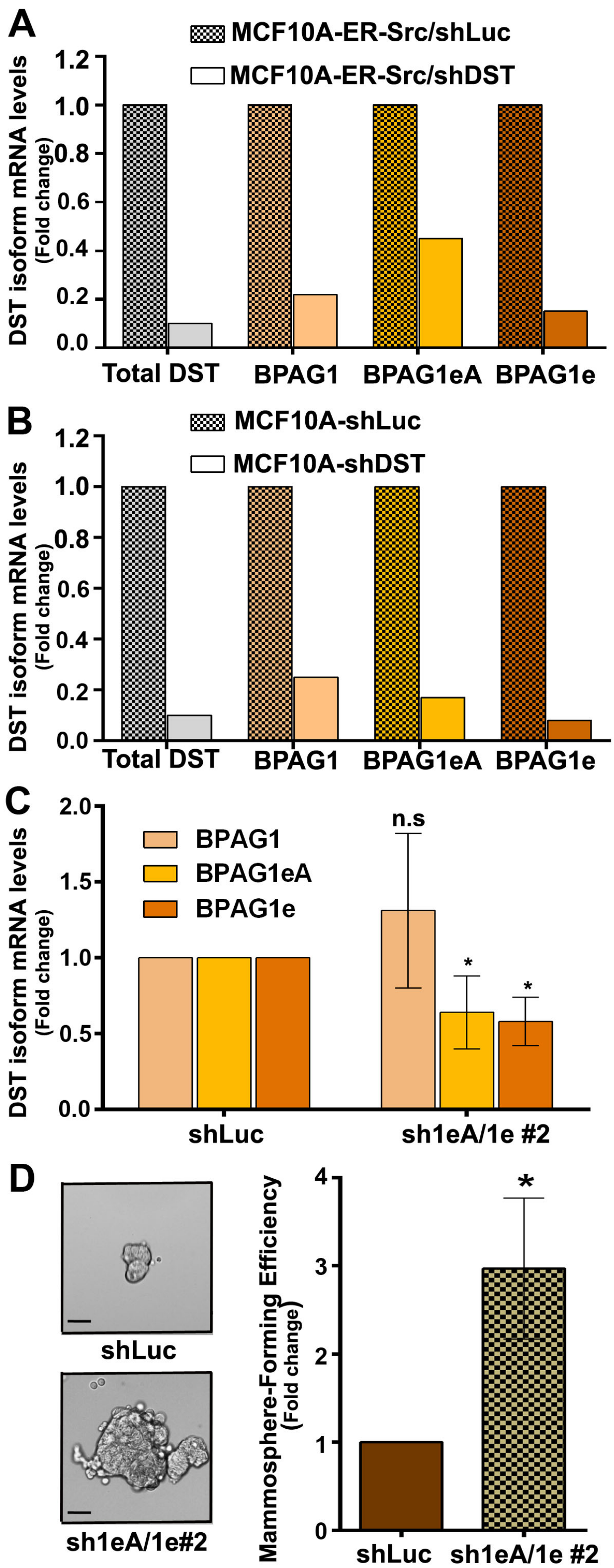


**Supplementary Figure S3: Expressing shBPAG1eA/1e affects BPAG1eA and BPAG1e expression specifically and promotes mammosphere formation (A)** Fold change of BPAG1 or BPAG1eA or BPAG1e mRNA levels between shLuc- or shDST-expressing MCF10A-ER-Src cells, normalized to GAPDH. **(B)** Fold change of BPAG1 or BPAG1eA or BPAG1e mRNA levels between shLuc- or shDST-expressing MCF10A cells, normalized to

GAPDH. **(C)** Fold change of BPAG1 or BPAG1eA or BPAG1e mRNA levels between shLuc- and shBPAG1eA/1e#2-expressing MCF10A cells, normalized to GAPDH. Data are from three biological replicates, performed in triplicates. **(D)** (Left panels) Representative images of shLuc- or shBPAG1eA/1e#2-expressing MCF10A mammospheres. Scale bars represent 50 μm. (Right panel) Fold change in mammosphere-forming efficiency between shLuc- and shBPAG1eA/1e#2-expressing MCF10A cells. Data are from three biological replicates, performed in triplicates. For all quantifications, error bars indicate SD.; ns indicates non-significant; * indicates $P<0.05$.

## Supplementary Tables

| | | |
|---|---|---|
| shDST | GCTTATGACTGGAGTGAGA | All DST Isoforms |
| shDST # 2 | AGAGAAAGATTCAGGAAAA | All DST Isoforms |
| shBPAG1eA/1e | ACCGTTAGAAGTAGAGCTT | BPAG1eA ad BPAG1e |
| shBPAG1eA/1e # 2 | CCTCCTACCTTTTAGTCTA | BPAG1eA ad BPAG1e |
| shLuciferase | AACGTACGCGGAATACTTC | Non-targeting |

**Supplementary Table S1:** shRNA sequences used in this study

| | | |
|---|---|---|
| **Replicate No.** | **MCF10A-shLuc** | **MCF10A-shDST** |
| 1 | 8.75 | 10.75 |
| 2 | 13.50 | 14.30 |
| 3 | 11.25 | 10.00 |
| 4 | 13.25 | 15.75 |
| **Average ± S.D.** | **11.69 ± 2.20** | **12.70 ± 2.77** |

**[p-value > 0.05; Paired t-test - Statistically non-significant]**

**Supplementary Table S2:** Cell number quantification 72 hours after treatment of MCF10A-shLuc and MCF10A-shDST cells with Tet for evaluating YAP sub-cellular localization and the expression of YAP target genes.

| | | |
|---|---|---|
| GAPDH forward | CTCTGCTCCTCCTGTTCGAC | Ensemble Release 80 |
| GAPDH reverse | ACCAAATCCGTTGACTCCGAC | |
| Total DST forward | GATGCAGATCCGAAAACCCCT | Ensembl Release 85 |
| Total DST reverse | CTCAGTGCGGTCCAGTTGTA | |
| Long DST Isoforms (BPAG1) forward | CCACAGGCCACACACTTCTT | Ensembl Release 80 |
| Long DST Isoforms (BPAG1) reverse | GGGATGCGGAGCCAGATTT | |
| BPAG1eA forward | TGTTGAAGCATATTTAAGGAGCCAC | Ensemble Release 80 |
| BPAG1eA reverse | AGGTAGGAGGTCTGGAAAAAGC | |
| BPAG1e forward | TCAGGGCAGCAATATCAGTG | Ensemble Release 87 |
| BPAG1e reverse | ACTAACCGGCTCAGCAAAGA | |
| CTGF forward | CTCGCGGCTTACCGACTG | NCBI Release 105 |
| CTGF reverse | GGCTCTGCTTCTCTAGCCTG | |
| CYR61 forward | CCTTGTGGACAGCCAGTGTA | NCBI Release 105 |
| CYR61 reverse | ACTTGGGCCGGTATTTCTTC | |
| ITGB6 forward | AATCGGTCTGCACAGCAAGA | Ensemble Release 94 |
| ITGB6 reverse | ACAGCCACCTTGTACGTGAT | |
| Cyclin D1 forward | GATCAAGTGTGACCCGGACTG | NCBI Release 105 |
| Cyclin D1 reverse | CCTTGGGGTCCATGTTCTGC | |
| Zyxin forward | AGAAGAAGTTCGGCCCTGTG | Ensemble Release 94 |
| Zyxin reverse | GGAGGCAGGGGAAAGTCTTC | |

**Supplementary Table S3:** Primers used for qRT-PCR.

| | | |
|---|---|---|
| **Ensembl Release 95** | **NCBI Release 109** | **In this study** |
| DST-207 (ENST00000421834.6) | Isoform 1 (NM_183380) | Not included |
| DST-202 (ENST00000312431.10) | Isoform 2 (NM_001144769.2) | BPAG1 |
| | Isoform 3 (NM_001144770.1) | |
| DST-201 (ENST00000244364.10) | Isoform 1eA (NM_015548) | BPAG1eA |
| DST-205 (ENST0000030765.10) | Isoform 1e (NM_001723) | BPAG1e |

**Supplementary Table S4**: DST isoforms nomenclature and accession numbers in Ensembl (Release 95), NCBI (Release 109) and in this study.
